# Platelet Concentrates in Periodontal Regeneration: A Review of Current Evidence and Clinical Perspectives

**DOI:** 10.7759/cureus.113341

**Published:** 2026-07-25

**Authors:** Vartika Verma, Ravi Kunnaiah, Md Kafeel Ahmed, Aamir Shamas, Meghna Pujara, Subha S Dany, Rubeena Naaz, Neena Bhatti

**Affiliations:** 1 Periodontology and Oral Implantology, Kalka Dental College, Meerut, IND; 2 Periodontology, MES Dental College, Perinthalmanna, IND; 3 Periodontology, MNR Dental College and Hospital, Sangareddy, IND; 4 Periodontology, Ministry of Health, Specialized Dental Center, Turaif, SAU; 5 Periodontology, Faculty of Dental Science, Dharmsinh Desai University, Nadiad, IND; 6 Public Health Dentistry, Hi-Tech Dental College and Hospital, Bhubaneswar, Bhubaneswar, IND; 7 Dentistry, SRK Dental Clinic, Hyderabad, IND; 8 Pharmacology and Therapeutics, Christian Medical College and Hospital, Ludhiana, IND

**Keywords:** growth factors, intrabony defects, periodontal regeneration, platelet concentrations, platelet-rich fibrin, platelet-rich plasma

## Abstract

Periodontitis is a chronic inflammatory disease characterized by irreversible destruction of the periodontal ligament, cementum, and alveolar bone, making true periodontal regeneration a significant therapeutic challenge. Autologous platelet concentrates have emerged as promising biological adjuncts because they provide a fibrin scaffold enriched with platelets, leukocytes, and growth factors that promote angiogenesis, cell proliferation, extracellular matrix synthesis, and wound healing. This review summarizes the biological basis, classification, mechanisms of action, and current clinical evidence regarding platelet-rich plasma (PRP), platelet-rich fibrin (PRF), advanced platelet-rich fibrin (A-PRF), injectable platelet-rich fibrin (i-PRF), and titanium-prepared platelet-rich fibrin (T-PRF) in periodontal regeneration. Current evidence indicates that PRF is the most extensively investigated preparation and demonstrates consistent improvements in probing pocket depth, clinical attachment level, and radiographic bone fill, particularly in periodontal intrabony defects when used as an adjunct to open flap debridement or bone grafting; a recent network meta-analysis reported statistically significant standardized improvements in all three outcomes favoring PRF-based therapy. Evidence supporting the use of platelet concentrates in furcation defects, gingival recession, periodontal plastic surgery, and extraction socket preservation is encouraging but remains limited by heterogeneity in study design and preparation protocols. Overall, platelet concentrates represent valuable adjuncts to regenerative periodontal therapy; however, standardized preparation methods and well-designed multicenter randomized controlled trials are required to establish the comparative effectiveness of newer platelet concentrate preparations and optimize their clinical application.

## Introduction and background

Periodontitis is a chronic inflammatory disease driven by dysbiotic subgingival biofilm and the host immune response, leading to irreversible destruction of the periodontal ligament, cementum, and alveolar bone [1]. Conventional periodontal therapy arrests disease progression, but the healed site typically exhibits a long junctional epithelium or connective tissue adaptation rather than true regeneration. True periodontal regeneration demands the simultaneous formation of new cementum, new alveolar bone, and functionally oriented periodontal ligament fibers - a process that is biologically demanding and technique-sensitive [2].

Established regenerative strategies include open flap debridement (OFD), guided tissue regeneration (GTR) with barrier membranes, bone grafts, enamel matrix derivatives, and autologous platelet concentrates [2]. Outcomes remain case-specific and are influenced by defect morphology, wound stability, plaque control, smoking, systemic health, and maintenance compliance [1,2].

Autologous platelet concentrates have attracted interest because activated platelets release growth factors that stimulate cell migration, matrix synthesis, and angiogenesis, while the resulting fibrin provides a physical scaffold that stabilizes the wound and delivers these mediators locally [3,4]. They are commonly grouped by generation: first-generation platelet-rich plasma (PRP), which requires anticoagulants and exogenous activation; second-generation platelet-rich fibrin (PRF), prepared without anticoagulants to yield a natural fibrin clot; and subsequent PRF derivatives developed to modify cellular content, handling, or hemocompatibility - advanced PRF (A-PRF), injectable PRF (i-PRF), and titanium-prepared PRF (T-PRF). Their autologous origin, chairside preparation, and dual role as a growth factor reservoir and scaffold make them attractive adjuncts to periodontal surgical procedures [5,6].

Although PRF has become the most widely investigated preparation, the evidence base is uneven: robust data exist for intrabony defects, whereas comparative evidence for PRP and for the newer A-PRF, i-PRF, and T-PRF preparations is more limited, and variability in centrifugation protocols continues to hinder direct comparison across studies [2,5]. With the introduction of newer platelet concentrate preparations and the growing body of clinical evidence, an updated review is needed to summarize current knowledge, identify areas of consensus, and highlight existing gaps in the literature. This review therefore synthesizes the biological mechanisms, classification, clinical applications, and current evidence for platelet concentrates in periodontal regeneration; clarifies which indications are presently well supported; and identifies where standardized protocols and higher-quality trials are most needed.

## Review

Search strategy

This article is a review of the literature and was not designed or reported as a systematic review. Therefore, it does not include a PRISMA flow diagram, a formal risk-of-bias assessment, or quantitative pooling of data by the present authors. Electronic databases (PubMed/MEDLINE, the Cochrane Library, Scopus, and Google Scholar) were searched from inception through March 2026 using combinations of the terms “platelet-rich plasma,” “platelet-rich fibrin,” “advanced platelet-rich fibrin,” “injectable platelet-rich fibrin,” “titanium-prepared platelet-rich fibrin,” “periodontal regeneration,” “intrabony defect,” “furcation defect,” and “gingival recession.” Reference lists of eligible systematic reviews and meta-analyses were manually screened to identify additional relevant studies. Two authors independently screened titles, abstracts, and full-text articles for eligibility, and disagreements were resolved by discussion and consensus. English-language human clinical studies, randomized controlled trials, systematic reviews, meta-analyses, and biologically relevant experimental studies were considered eligible, whereas conference abstracts, editorials, letters, and duplicate publications were excluded. As this is a review of the literature, completeness of the available evidence cannot be guaranteed, and the studies discussed reflect the current distribution of published evidence, which is more extensive for PRF than for PRP or the newer A-PRF, i-PRF, and T-PRF preparations.

The descriptors of evidence strength used in the text (e.g., “strongest available,” “moderate,” and “emerging”) represent a qualitative appraisal based on the number, consistency, and methodological design of the available studies for each clinical indication. They do not represent a formal evidence-grading system such as the Grading of Recommendations Assessment, Development and Evaluation (GRADE) and should be interpreted accordingly.

Biological basis of platelet concentrates

Platelet Biology and Growth Factor Release

Platelets respond immediately to tissue injury by forming a hemostatic plug and releasing bioactive mediators from alpha granules, including platelet-derived growth factor (PDGF), transforming growth factor-beta (TGF-β), vascular endothelial growth factor (VEGF), insulin-like growth factor (IGF), and epidermal growth factor (EGF) [3,4]. These mediators collectively drive cell migration, proliferation, matrix synthesis, and angiogenesis [4]. The biological activity of any platelet concentrate depends on platelet concentration, fibrin architecture, growth factor release kinetics, leukocyte content, and the local wound environment [5]. Table 1 summarizes the major growth factors and their specific relevance to periodontal regeneration [3,4].

**Table 1:** Major growth factors released by platelets and their periodontal relevance PDGF: platelet-derived growth factor; TGF-β: transforming growth factor-beta; VEGF: vascular endothelial growth factor; IGF: insulin-like growth factor; EGF: epidermal growth factor Adapted and synthesized by the authors from [3,4].

Growth factor	Main biological role	Relevance in periodontal regeneration
PDGF	Fibroblast migration, proliferation, collagen synthesis	Connective tissue repair; periodontal ligament healing
TGF-β	Extracellular matrix production; cellular differentiation	Tissue maturation; inflammatory regulation
VEGF	Angiogenesis; endothelial cell migration	Vascular supply to regenerating defects
IGF	Cell proliferation; matrix synthesis	Soft tissue and bone healing support
EGF	Epithelial migration; soft tissue closure	Wound coverage; early epithelial repair

Fibrin Matrix as a Biological Scaffold

During centrifugation without anticoagulants, PRF forms a three-dimensional fibrin network that stabilizes the wound clot, supports fibroblast and endothelial cell migration, and enables gradual growth factor release over time [6]. This progressive release matters because periodontal regeneration depends on a coordinated healing sequence rather than a single biological stimulus, and early clot disruption from flap movement or bacterial contamination can prevent new attachment formation [2,6].

Immunomodulatory Effects

Platelet concentrates modulate local inflammatory activity, and leukocyte-containing preparations such as leukocyte- and platelet-rich fibrin (L-PRF) may provide additional antimicrobial defense through leukocyte-platelet interactions [7]. These effects complement, but do not replace, thorough debridement and infection control as the prerequisite for any regenerative procedure [1,7].

Classification of platelet concentrates

Platelet concentrates are classified by preparation method, anticoagulant use, fibrin architecture, and cellular composition, since each preparation has distinct biological and mechanical properties that influence clinical behavior [5].

Platelet-Rich Plasma (PRP)

PRP is a first-generation concentrate prepared from anticoagulated venous blood and requires exogenous activation with thrombin or calcium chloride. It is generally prepared using a two-step centrifugation protocol to concentrate platelets before activation, although centrifugation parameters vary among commercially available systems. It can be applied as a liquid or gel together with bone grafts and barrier membranes [3]. Variable platelet yields and reliance on activators have driven the adoption of fibrin-based preparations in periodontal practice [5].

Platelet-Rich Fibrin (PRF)

PRF is prepared by immediate centrifugation of freshly collected venous blood without anticoagulants, commonly using a single-spin protocol. Centrifugation speed, relative centrifugal force, duration, rotor design, and tube material vary between systems and influence the biological characteristics of the final clot [5]. It can be compressed into membranes, placed into defects, or mixed with graft particles, and is the most widely applied platelet concentrate in periodontal regeneration [8].

Advanced Platelet-Rich Fibrin (A-PRF)

A-PRF is prepared using lower relative centrifugal force and a longer centrifugation time than conventional PRF, enhancing the retention of leukocytes and growth factors within the fibrin matrix. Clinical superiority over conventional PRF has not yet been confirmed in adequately powered comparative trials [7].

Injectable Platelet-Rich Fibrin (i-PRF)

It is prepared using very low-speed, short-duration centrifugation to obtain a liquid formulation before fibrin polymerization occurs. The resulting cohesive, moldable mass termed “sticky bone” may improve graft handling and reduce particle migration within irregular defects [9].

Titanium-Prepared Platelet-Rich Fibrin (T-PRF)

**Figure 1:**
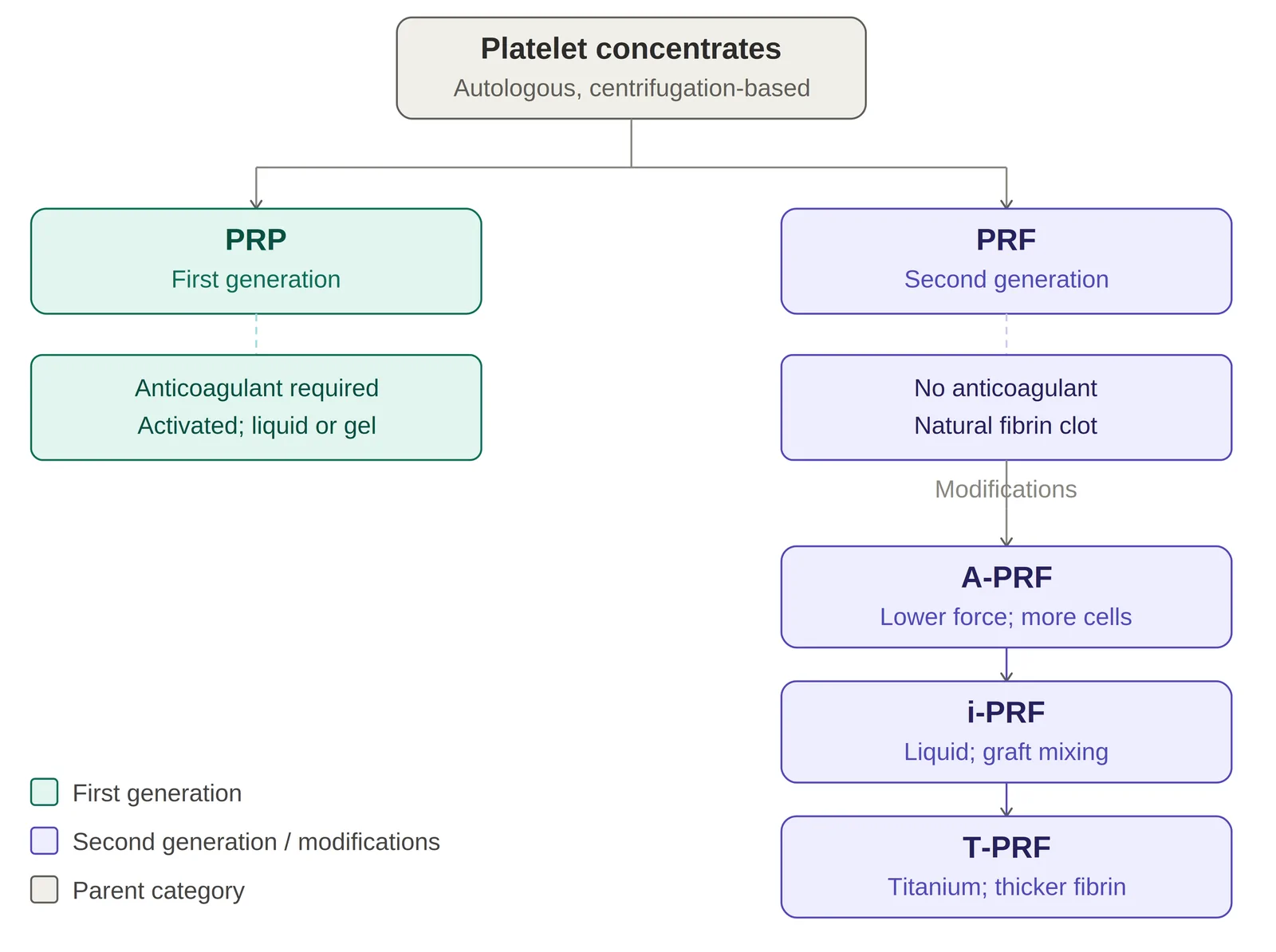
Classification of platelet concentrates used in periodontal regeneration PRP: platelet-rich plasma, PRF: platelet-rich fibrin, A-PRF: advanced PRF, i-PRF: injectable PRF, T-PRF: titanium-prepared PRF The figure was created by the authors (Vartika Verma, Ravi Kunnaiah, Kafeel Ahmed) using Microsoft PowerPoint (Microsoft Corporation, Redmond, WA, USA).

T-PRF follows a preparation protocol similar to conventional PRF but uses titanium tubes instead of glass tubes, which may influence fibrin architecture and biocompatibility. Clinical evidence for T-PRF in periodontal regeneration is limited, and randomized trials are needed before superiority over standard PRF can be established [10]. The classification hierarchy and key characteristics of platelet concentrates used in periodontal practice are illustrated in Figure 1 [5,7-9,11].

Mechanisms of action in periodontal regeneration

Soft Tissue Healing and Angiogenesis

Following periodontal surgery, the fibrin scaffold stabilizes the wound clot while released growth factors promote fibroblast migration, collagen production, and soft tissue repair, and VEGF-driven angiogenesis supplies oxygen, nutrients, and progenitor cells to the regenerating defect [3,4,6].

Osteogenesis and New Attachment Formation

Platelet concentrates support osteoblast migration, proliferation, and differentiation, and may improve early graft stability when combined with bone grafts [6]. Radiographic bone fill, however, does not confirm true periodontal regeneration; histological evidence of new cementum and functionally oriented periodontal ligament fibers is required but is rarely verifiable in human clinical studies [8].

Synergistic Role With Bone Grafts and Membranes

When combined with bone grafts or barrier membranes, platelet concentrates serve a complementary rather than a replacement role: graft particles provide osteoconductive space maintenance, the fibrin component improves cohesion and local growth factor retention, and membranes limit epithelial downgrowth [11,12]. Platelet concentrates cannot substitute for sound surgical execution, adequate root surface preparation, defect containment, and primary wound closure, all of which remain essential determinants of the regenerative outcome [12,13].

Clinical applications and outcomes

Intrabony Defects

Intrabony defects represent the indication with the best-supported evidence base for platelet concentrates. Deep, narrow, and well-contained defects are most favorable because residual bony walls preserve blood supply and clot stability [2,8]. A recent systematic review and network meta-analysis of randomized controlled trials reported statistically significant standardized improvements favoring PRF-based therapy, with overall standardized mean differences of approximately 0.59 for probing depth reduction, 0.67 for clinical attachment level (CAL) gain, and 1.54 for radiographic bone fill (all p < 0.01) [8]. These benefits were most consistent when PRF was combined with OFD or bone grafts [8,13,14].

Furcation Defects

Furcation defects are more challenging than intrabony defects due to complex root anatomy, limited instrumentation access, and narrow furcation entrances [1]. PRF has been evaluated with OFD, grafts, and membranes in Class II furcation defects, with some evidence of benefit; however, available studies are fewer and more heterogeneous than those for intrabony defects, and careful case selection remains essential [8,15].

Gingival Recession and Root Coverage

PRF membranes used with the coronally advanced flap (CAF) may improve soft tissue healing, flap adaptation, and postoperative comfort while reducing donor-site morbidity by avoiding palatal graft harvesting [8,16]. Connective tissue grafting remains more predictable for long-term root coverage and tissue thickening, and PRF should not be considered a universal substitute in all clinical situations [8,16].

Periodontal Plastic Surgery and Extraction Sockets

In periodontal plastic surgery, PRF membranes and i-PRF may support gingival augmentation, flap healing, and soft tissue maturation, with outcomes dependent on flap design, tissue phenotype, and tension-free closure [8,9]. In extraction sockets, PRF may support clot stabilization and accelerate soft tissue closure, though its role in preventing ridge resorption depends on socket integrity, infection status, and biomaterial selection [8,17]. The clinical indications, preparations used, reported benefits, and current strength of evidence for platelet concentrates in periodontal regeneration are summarized in Table 2 [13-17].

**Table 2:** Clinical applications and reported outcomes of platelet concentrates in periodontal regeneration *Strength of evidence reflects a narrative appraisal based on the number, consistency, and design of available studies; it was not derived from a formal grading system such as GRADE. PRF: platelet-rich fibrin; A-PRF: advanced PRF; i-PRF: injectable platelet-rich fibrin; OFD: open flap debridement; CAF: coronally advanced flap; PPD: probing pocket depth; CAL: clinical attachment level; GRADE: Grading of Recommendations Assessment, Development and Evaluation Adapted and synthesized by the authors from [13-17].

Clinical indication	Preparation used	Reported benefit	Strength of current evidence*
Intrabony defects	PRF; i-PRF; PRF + graft	PPD reduction; CAL gain; radiographic bone fill	Strongest available
Class II furcation defects	PRF + OFD or graft	Improved healing in selected cases	Moderate to limited
Gingival recession	PRF/A-PRF membrane + CAF	Root coverage; reduced donor-site morbidity	Moderate
Periodontal plastic surgery	PRF; i-PRF	Soft tissue maturation; patient comfort	Emerging
Extraction sockets	PRF	Clot stabilization; soft tissue closure	Moderate

Assessment of clinical outcomes

Probing Pocket Depth (PPD) and Clinical Attachment Level (CAL)

PPD reflects the inflammatory status of the periodontium, with persistent deep pockets indicating a higher risk of recurrence [1]. CAL is the more meaningful regenerative parameter because it quantifies attachment recovery relative to a fixed anatomical landmark and is less susceptible to gingival margin changes [8]. Both measures should be interpreted alongside radiographic bone changes and long-term soft tissue stability [2,8].

Radiographic Bone Fill

Conventional periapical radiographs provide two-dimensional assessment of intrabony defect depth and bone density, while cone-beam computed tomography (CBCT) offers three-dimensional volumetric evaluation. CBCT may be justified in selected complex cases but is not routinely recommended given radiation exposure, cost, and inter-study protocol variability; it should complement rather than replace standard clinical measurements [18].

Soft Tissue and Patient-Reported Outcomes

Root coverage outcomes encompass percentage and complete root coverage, keratinized tissue width, gingival thickness, and esthetic integration [8,16]. Patient-centered outcomes - including pain, swelling, time to wound closure, and overall satisfaction - are increasingly recognized as essential complements to clinical measurements, particularly when comparing procedures with and without autologous materials [8,17].

Factors influencing clinical success

Preparation-Related Factors

The type of platelet concentrate determines fibrin architecture, leukocyte content, and growth factor release kinetics, all of which affect biological behavior within a defect [5,7]. Centrifugation speed, duration, rotor geometry, tube type, and blood volume substantially influence cell distribution and growth factor yield; lower relative centrifugal force improves cell retention and growth factor release in i-PRF preparations. Standardized and validated centrifugation protocols are therefore essential for clinical reproducibility and for meaningful cross-study comparison [5].

Defect and Surgical Factors

Defect morphology is a primary determinant of regenerative predictability, and pre-surgical assessment of containment and wall number is essential before selecting the regenerative approach [2,8]. One-wall and non-contained defects often require additional graft or membrane support [2]. Flap design, papilla preservation, root surface debridement, and primary wound closure are equally critical; minimally invasive surgical techniques improve wound stability and reduce postoperative complications [13].

Patient-Related Factors

Oral hygiene, smoking, glycemic control, systemic health, and maintenance compliance are strong determinants of regenerative outcomes [1]. Current evidence does not demonstrate a consistent effect of patient sex on the biological characteristics or clinical effectiveness of PRF, although further studies are needed to clarify any potential gender-related differences [6]. Smoking impairs angiogenesis and immune defense, while uncontrolled diabetes prolongs the inflammatory phase; comprehensive pre-surgical risk assessment and optimization of modifiable risk factors are therefore essential before selecting regenerative therapy [1].

Advantages and Current Challenges

**Figure 2:**
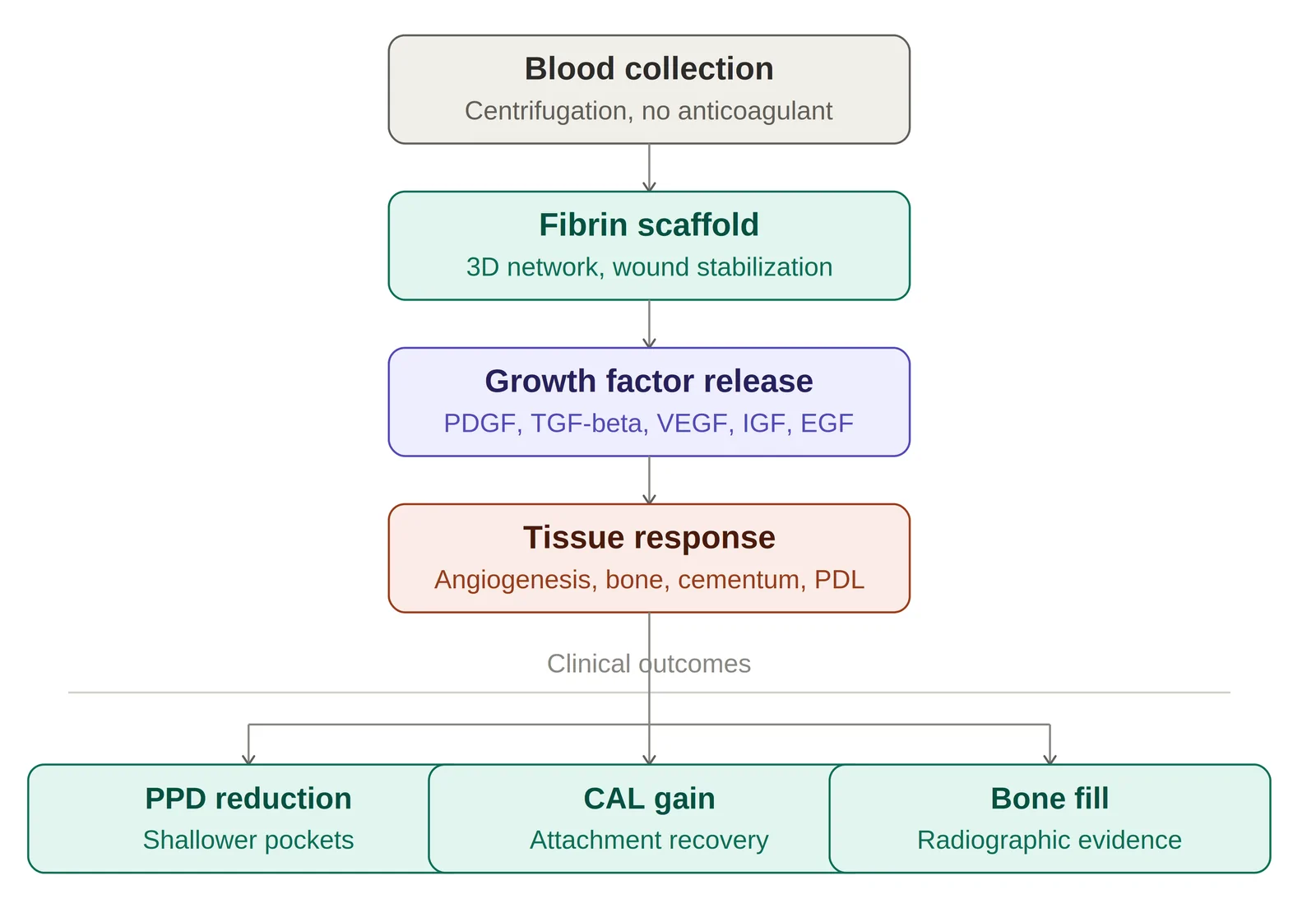
Mechanism of action of platelet concentrates in periodontal regeneration PDGF: platelet-derived growth factor; TGF-β: transforming growth factor-beta; VEGF: vascular endothelial growth factor; IGF: insulin-like growth factor; EGF: epidermal growth factor; PDL: periodontal ligament; PPD: probing pocket depth; CAL: clinical attachment level The figure was created by the authors (Aamir Shamas, Meghna Pujara, Subha Dany, Rubeena Naaz) using Microsoft PowerPoint (Microsoft Corporation, Redmond, WA, USA).

The principal advantages of platelet concentrates are their autologous origin, which eliminates immunogenic risk and disease transmission concerns, and their established role as both a growth factor delivery system and a fibrin scaffold across multiple periodontal indications [5,8]. Their most significant limitation is the absence of universal preparation protocols; centrifugation parameters and tube materials differ substantially across studies, which, together with the sparse long-term data for A-PRF, i-PRF, and T-PRF, precludes direct comparison and is examined further in the Limitations section [5,7,9,10]. The sequential mechanism by which platelet concentrates support periodontal regeneration, from blood collection through fibrin scaffold formation and growth factor release to measurable clinical outcomes, is illustrated in Figure 2 [3,4,6,8].

Limitations

This review has several limitations. First, it is a review of the literature and therefore does not follow the rigorous methodology of a systematic review or meta-analysis, introducing the potential for selection bias; although multiple electronic databases were searched using a structured strategy, relevant studies published in languages other than English or indexed outside the selected databases may not have been captured. Second, the available literature demonstrates substantial heterogeneity in study design, patient populations, periodontal defect morphology, surgical techniques, centrifugation protocols, and outcome measures, limiting direct comparison across studies. Many investigations also have relatively small sample sizes and short follow-up periods, reducing the ability to draw definitive conclusions regarding long-term clinical effectiveness. While PRF has the most robust supporting evidence, high-quality comparative data for newer preparations such as A-PRF, i-PRF, and T-PRF remain limited. Finally, most clinical studies rely on surrogate clinical and radiographic outcomes, whereas histological confirmation of true periodontal regeneration is rarely feasible in human studies.

Future directions

Future research should prioritize well-designed, multicenter randomized controlled trials using standardized preparation protocols to enable meaningful comparisons among different platelet concentrate preparations. Uniform reporting of centrifugation parameters, relative centrifugal force, tube materials, platelet and leukocyte concentrations, and growth factor release profiles would improve reproducibility and facilitate evidence synthesis. Long-term studies evaluating the comparative effectiveness of PRP, PRF, A-PRF, i-PRF, and T-PRF across different periodontal defect morphologies are needed to establish evidence-based clinical recommendations.

Emerging regenerative strategies combining platelet concentrates with stem cell therapy, tissue-engineered scaffolds, bioactive biomaterials, and three-dimensional printing technologies warrant further investigation [19]. Future studies should also incorporate patient-reported outcome measures, esthetic outcomes, cost-effectiveness analyses, and quality-of-life assessments alongside conventional clinical parameters. Advances in artificial intelligence and digital imaging may further improve treatment planning, radiographic assessment, and individualized regenerative approaches, contributing to more precise and predictable periodontal regeneration.

## Conclusions

The biological rationale for platelet concentrates in periodontal regeneration growth factor release, fibrin scaffolding, angiogenic support, and immune modulation aligns well with the requirements of coordinated periodontal wound healing. In practice, this translates most consistently into benefit for intrabony defects, where PRF, particularly as an adjunct to OFD or bone grafting, improves PPD, CAL, and radiographic bone fill. Evidence for furcation defects, gingival recession, and periodontal plastic surgery is promising but heterogeneous, and the comparative efficacy of A-PRF, i-PRF, and T-PRF remains insufficiently characterized. Until standardized preparation protocols and adequately powered multicenter randomized controlled trials become available, platelet concentrates are best regarded as valuable biological adjuncts rather than stand-alone regenerative therapies.
